# Comparison of Psychological and Cognitive Characteristics between Professional Internet Game Players and Professional Baseball Players

**DOI:** 10.3390/ijerph17134797

**Published:** 2020-07-03

**Authors:** Jin Oh Kang, Kyoung Doo Kang, Jea Woog Lee, Jae Jun Nam, Doug Hyun Han

**Affiliations:** 1 Department of Psychiatry, College of Medicine, Chung-Ang University, Seoul 06973, Korea; kjo88815@naver.com (J.O.K.); cad344@gmail.com (K.D.K.); 2 Department of Information & Technology in Sport, College of Sport Science, Chung-Ang University, Seoul 06974, Korea; yyizeuks@cau.ac.kr; 3 Department of Golf, Korea Golf University, Hoeng Seong, Gang won-do 25247, Korea; 01197097765@nate.com

**Keywords:** esports players, pro-baseball players, character, temperament, anxiety, cognitive functions

## Abstract

The esports industry is increasing in popularity and is now played at the professional level. We hypothesized that esports players may have a significant advantage over the general population in terms of psychological and cognitive characteristics, which may be similar to that of professional baseball players. We recruited three participant groups: esports players (*n* = 55), pro-baseball players (*n* = 57), and age- and sex-matched healthy comparison subjects (*n* = 60). We assessed psychological status using the Korean versions of Temperament and Character Inventory and State and Trait Anxiety Inventory and cognitive functions using the modified Tower of London, Emotional Perception, and Mental Rotation tests. Esports players had similar psychological characteristics to pro-baseball players (higher novelty seeking [*p* < 0.01 *, ŋ = 0.818], self-directedness [*p* < 0.01 *, ŋ = 0.757], and self-transcendence scores [*p* < 0.01 *, ŋ = 0.853], and decreased state anxiety scores [*p* < 0.01 *, ŋ = 0.808]), which differed from those of the general population. However, esports players showed higher working memory [*p* < 0.01 *, ŋ = 0.823] and slower emotional perception than pro-baseball players [*p* < 0.01 *, ŋ = 0.812]. In conclusion, esports and pro-baseball players had similar psychological but different cognitive characteristics.

## 1. Introduction

### 1.1. Esports Players and Professional Baseball Players

The worldwide gaming industry is growing daily, and esports are a big part of it. The International Esports Federation (IeSF) was launched in 2008, and the competition rules and player management have since been systematized. Since esports are increasing in popularity, they are now played at a professional level [1]. Esports players are trained systemically in a team, receive an annual salary, and participate in three–five pro-gaming leagues every year. Each pro-team has one general manager, two to four coaches, and several other staff members. As esports are now discussed alongside regular sports, esports players are considered professionals and experts in their field. Recently, esports was selected as the demonstration competition at the Jakarta Palembang Asian Games of 2018, organized by the Asian Electronic Sport Federation (AESF). The event was successful and is expected to be adopted as a formal event at the Hangzhou Asian Games of 2022.

Professional baseball is one of the most representative professional sports in Korea, which as of 2020 has 10 professional baseball teams. Professional baseball players are trained systemically in a team, receive an annual salary, and participate in one pro-baseball league per year. Each team has two general managers, 20–30 coaches, 50–60 other managerial staff members, and around 90 players.

Although esports is an officially accepted professional sport, many still feel that esports players should not be recognized as athletes, believing that esports professionals are no different from amateur players who just enjoy the game or are simply addicted to gaming [2]. Many research studies on internet gaming have focused on problematic internet game playing, and only a few clinical studies on internet gaming were interested in the cognitive effects of internet game playing. Several studies suggested that the cause of problematic internet game play is associated with psychological characteristics including temperament [3,4]. Our aim was to qualify esports players as professional sports players in terms of their psychological and cognitive characteristics by comparing them to the most popular professional sports players (i.e., professional baseball players).

### 1.2. Psychological and Cognitive Characteristics of Esports Players and Professional Baseball Players

In studies examining the psychological characteristics of internet gaming players using the Temperament and Character Inventory (TCI), internet players demonstrated higher scores in novelty seeking than comparison subjects who did not play internet game [3,4]. However, there have been no TCI studies of esports players. Esports game play is thought to engage several cognitive domains including attention, perception and information processing, visuo-spatial working memory, and hand-eye coordination [5,6,7,8,9,10,11]. In addition, emotion regulation during play was reported to be an essential and necessary skill [12]. Several studies have suggested that esports players also have excellent working memory abilities [13,14]. Han et al. [13] reported that esports players required fewer trials and total errors to complete six categories of the Wisconsin Card Sorting Test, a task assessing cognitive flexibility and task switching. Action video game players could detect changes in objects faster and recognize the belonging of stimuli to their respective categories earlier, owing to enhanced perpetual analysis abilities than comparison subjects who did not play internet games [14].

In several studies involving professional sports players, the Korean version of the TCI [15] and the Korean version of the State-Trait Anxiety Inventory-Y (STAI-KY) were used to describe the psychological characteristics of athletes such as soccer, baseball, and basketball players [16]. In the comparison of two groups of national pro-basketball players, Japanese pro-basketball players showed higher scores of self-directedness and cooperativeness than Korean pro-basketball players [17]. Korean major league baseball players showed increased scores of novelty seeking and reward dependence than healthy general people [18]. In addition, the state anxiety scores were negatively correlated with the novelty seeking scores in the Korean major league baseball players [18]. The anxiety levels of athletes, which can be measured using the STAI-KY, also have an important effect on performance decline [19]. The novelty seeking score was thought to be associated with active and challenging personality traits [15]. 

In addition to psychological characteristics, the cognitive aspects of athletes can be assessed using the Tower of London test, the Emotional Perception test, and the Mental Rotation test, which measure working memory, intuitive perception, and time-space perception ability, respectively. These faculties are critical in the ability and performance of an individual athlete and many studies have focused on these three cognitive aspects in athletes [20,21,22].

### 1.3. Hypotheses

In the current study, we aimed to qualify esports players as professional sports players in terms of their psychological and cognitive characteristics. With respect to novelty seeking, we hypothesized that esports players and professional baseball players might show similar psychological characteristics, such as high novelty seeking, compared to the general population. We also hypothesized that esports players would show better working memory than pro-baseball players and the general population.

## 2. Materials and Methods

### 2.1. Participants

We recruited 55 professional gamers (esports players), 57 professional baseball (pro-baseball) players, and 60 age- and sex-matched healthy comparison subjects from the general population. All participants were selected for recruitment by simple random sampling. Upon securing their permission, we visited professional esports and baseball teams. After explaining the goal and methods of the study, all players voluntarily consented to participate in the current study. 

In May of 2019, all the esports players recruited for this study were on one of two professional teams in the Korean Esports Association (KeSPA). As these two professional gaming teams did not want their names to be revealed, we describe them as team A and team B. A total of 55 players (team A, *n* = 27; team B, *n* = 28) agreed to participate in our study. 

On team A, there were 12 League of Legends players, eight Overwatch players, and seven Battleground players. Of the 27 players on team A, seven players attended 85% of the games during the pro-gaming season and 20 players attended less than 30% of the games. On team B, there were eight League of Legends players, eight Overwatch players, and 12 StarCraft players. Of the 28 players on team B, five players attended 82% of the games during the pro-gaming season and 23 players attended less than 40% of the games. At the beginning of each game season, the head coach selected players; better players could participate in more games. Accordingly, we defined the elite players (*n* = 12) as those who attended more than 80% of the games throughout the entire 2018 season. The other players were classified into the “general gamer” group (*n* = 43).

Between November 2018 and February 2019, 57 players on one professional Korea Baseball Organization (KBO) team agreed to participate in our study. The “elite” group (*n* = 14) included players with 446 plate appearances or 144 innings over 144 major league games in the 2018 season (2018 Korean Baseball Rules, KBO). The others were classified into the “general baseball players” group (*n* = 43).

Through an advertisement posted online, 60 healthy comparison subjects were recruited. The inclusion criteria for the healthy comparison subjects were: (1) male gender, (2) age 19–23 years, (3) no history of playing baseball, and (4) internet gameplay time less than 30 h/week. To rule out excessive internet game play, we limited internet game playing time (i.e., <30 h/week), which has been suggested in previous studies [5].

The protocol of the present study was approved by the Chung-Ang University Hospital Institutional Review Board (10-065-10-18), and written informed consent was provided by all participants.

### 2.2. Measures

#### 2.2.1. Assessment of Psychological Characteristics

The Korean version of the TCI was used in the trait analysis [23]. The TCI is used to assess an individual’s personality, and consists of two parts: temperament and social character [15]. The inventory consists of 240 true or false statements that evaluate four dimensions of temperament (novelty seeking, harm avoidance, reward dependence, and persistence) and three dimensions of character (self-directedness, cooperativeness, and self-transcendence) [18]. The Cronbach’s α and test-retest reliability of the TCI have been previously reported as 0.77 and 0.81, respectively [15].

The STAI-KY was used to estimate state and trait anxiety levels among our participants [18]. This scale includes a total of 40 items: 20 that describe trait anxieties and 20 that describe state anxieties. The Cronbach’s α of the STAI-KY is 0.91 for state and 0.82 for trait anxieties [24]. State anxiety is a transient emotional state representing apprehension, nervousness, and physiological signs, such as changes in heart rate and respiration. Trait anxiety is thought of as the stable tendency to experience negative emotions such as fears, worries, and anxiety [25].

#### 2.2.2. Assessment of Neurocognitive Function

Neurocognitive functions in all participants were assessed using a modified Tower of London test (CNT-MBI^®^, Seoul, Korea), the Emotional Perception test, and the Mental Rotation test.

**Figure 1:**
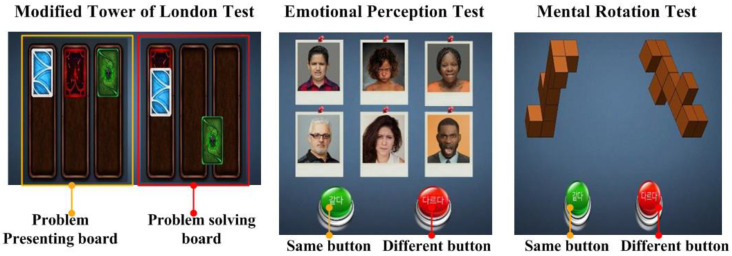
Tasks to assess cognitive functions.

The modified Tower of London test was used for the assessment of working memory, particularly to detect deficits in planning [26,27,28]. It consists of two boards with three lines and several cards of different colors. The computer uses the cards and lines to present the examinee with problem-solving tasks on the problem presenting board. The participant moves the cards on the problem-solving board to match the cards in the same order as presented on the problem presenting board. During the test, the number of moves of the cards and the reaction time were recorded. The test-retest reliability of the modified Tower of London test is 0.87 [26,27,28] (Figure 1). Faster reaction times and fewer moves in the Tower of London test represent better working memory [26,27,28].

The Emotional Perception test consisted of 108 questions, in which two to eight faces were presented at one time on the screen. The participants were asked to press the “same” or “different” buttons to indicate whether the faces depicted the same or different emotional expression. The possible emotional expressions were pleasant, neutral, and unpleasant, and were presented in different combinations on each trial. During the test, the mean correct rate and reaction time from presentation of the pictures to pushing of the buttons were recorded. The test-retest reliability for emotional face identification is 0.93 [29,30,31] (Figure 1). Faster reaction times and more correct responses in the Emotional Perception test represent better emotional perception [29,30,31].

In the Mental Rotation test, the computer presented a pair of three-dimensional (3D) objects, often rotated on a certain axis through a specific number of degrees (0°, 60°, 90°, 120°, or 180°). In some trials, the two 3D shapes presented were the same but rotated, and in other trials the shapes were different. The participants judged whether the two 3D objects were the same or different and pressed the “same” or “different” button to indicate their response. During the test, the mean correct rate and reaction time from presentation of the pictures to pushing of the buttons were recorded. Test-retest reliability is 0.91 [32,33,34] (Figure 1). Faster reaction times and more correct responses in the Mental Rotation test represent better spatio-temporal abilities [32,33,34].

### 2.3. Statistical Analysis

The demographic characteristics, psychological characteristics (TCI and anxiety), and neurocognitive functions (Tower of London test, Emotional Perception test, and the Mental Rotation test) among the esports players, pro-baseball players, and healthy comparison subjects were analyzed using one-way analysis of variance. To correct for multiple comparisons on the TCI, statistical significance was set at *p* < 0.013 (0.05/4) for the temperament domain (novelty seeking, harm avoidance, reward dependence, persistence) and at *p* < 0.017 (0.05/3) for the character domain (self-directedness, cooperativeness, self-transcendence). To correct for multiple comparisons on the STAI-KY (state anxiety and trait anxiety), statistical significance was set at *p* < 0.025 (0.05/2). To correct for multiple comparisons on cognitive functions (correction rate and reaction time), statistical significance was set at *p* < 0.025 (0.05/2).

Career years were compared between esports players and pro-baseball players using the independent *t*-test. For both esports and professional baseball, the psychological characteristics and neurocognitive functions were compared between elite players and general players using an independent *t*-test. Statistical significance was set at *p*-value < 0.05.

## 3. Results

### 3.1. Demographic Characteristics

There were no significant differences in age or education years between esports players, pro-baseball players, and healthy comparison subjects. In addition, there was no significant difference in career years between esports players and pro-baseball players. The esports players group consisted of 16 Overwatch players, seven Battleground players, 20 League of Legends players, and 12 StarCraft-2 players. The pro-baseball group consisted of 21 infield players, 12 outfield players, and 24 pitchers (Table 1).

### 3.2. Comparison of Psychological and Cognitive Characteristics between Esports Players, Pro-Baseball Players, and Healthy Comparison Subjects

In the TCI analysis, there were significant differences in novelty seeking, harm avoidance, self-directedness, and self-transcendence scores among the three groups. In the post hoc test, the esports players and pro-baseball players showed higher novelty seeking, self-directedness, and self-transcendence scores than healthy comparison subjects. Esports players had higher harm avoidance scores than the pro-baseball and healthy comparison groups (Table 2).

In the anxiety scales, esports players and pro-baseball players showed lower state anxiety scores than the healthy comparison subjects. However, there were no differences in trait anxiety among the groups (Table 2).

In the neurocognitive function tests, the esports players showed a faster reaction time in the Tower of London test and better performance (correct rate) on the Mental Rotation test than the pro-baseball and healthy comparison groups. In contrast, the pro-baseball group showed faster reaction times on the Emotional Perception and Mental Rotation tests than the esports players and healthy comparison subjects (Table 2).

### 3.3. Correlation between Psychological and Cognitive Characteristics in Esports Players, Pro-Baseball Players and Healthy Comparison Subjects

The self-transcendence scores were negatively correlated with reaction times on the Tower of London test in esports players (r = −0.44, *p* < 0.01) and with reaction times on the Emotional Perception test in pro-baseball players (r = −0.47, *p* < 0.01). There were no significant correlations between psychological and cognitive characteristics in the healthy comparison group.

### 3.4. Comparison of Psychological and Cognitive Characteristics between the Elite and General Groups

The elite esports players showed higher novelty seeking, self-directedness, and self-transcendence scores as well as lower state anxiety scores, faster reaction times on the Tower of London and Emotional Perception tests, and better performance on the Mental Rotation test than the general esports players (Table 3). Elite pro-baseball players showed higher scores on novelty seeking, self-directedness, and self-transcendence as well as lower scores on state anxiety and faster reaction times on the Emotional Perception and Mental Rotation tests than general pro-baseball players (Table 4).

## 4. Discussion

With respect to psychological characteristics, esports players and pro-baseball players were similar in their higher levels of novelty seeking, self-directedness, and self-transcendence as well as lower state anxiety. However, with respect to cognitive characteristics, there were several different trends between the two groups; esports players showed better executive function and spatio-temporal abilities, while professional baseball players showed better emotional perception.

### 4.1. Comparison of Psychological and Cognitive Characteristics between Esports Players, Pro-Baseball Players, and Healthy Comparison Subjects

Esports players and pro-baseball players showed higher novelty seeking scores than the healthy comparison group. Therefore, esports players and pro-baseball players are likely exploratory, curious, and passionate [15,16,35,36]. Novelty seeking is associated with exploratory excitement, openness, and extraversion as well as a sense of challenge [15,16,35]. People with these temperaments tend to pursue exciting adventures and enthusiastically search for new and unfamiliar situations. Thus, they find hidden rewards that others cannot anticipate [35,36]. They may try new things rather than just adapt to established rules or regulations [35,36], and this tendency can contribute to improving their gaming or baseball skills.

Furthermore, self-directedness and self-transcendence scores were high among esports and pro-baseball players. A high self-directedness score indicates great self-control, self-efficacy, and responsibility as well as a clear sense of purpose [15,16,37]; therefore, players with high self-directedness scores may be responsible and self-sufficient in their role in many gaming situations. Meanwhile, people with high self-transcendence have been regarded as patient and creative [15,16,38]; they may also be more likely to endure any ambiguities or uncertainties they face and be willing to accept failure maturely, even when they gave their best effort but still failed. While others may give up easily, individuals with a high self-transcendence score are likely to try repeatedly to improve their skills. These tendencies (high self-directedness and self-transcendence) are probably necessary for developing gaming skills at the professional level.

Conversely, esports players had significantly higher harm avoidance scores than pro-baseball players. People with higher harm avoidance scores usually prepare thoroughly when they expect risk, and this preliminary planning and preparation can help when they do face risks [15,16,39]. We think that individuals with higher harm avoidance may have “gamer” characters. Indeed, several studies using the TCI have suggested that internet gamers have higher harm avoidance than the general population [40]. During game playing, preliminary planning and preparation for battle may be associated with better performance.

In the STAI-KY, the anxiety traits of esports players were similar to those of the general population, but their state anxiety was lower, just like pro-baseball players. In other words, there was no significant difference in the characteristic anxiety they normally felt, but the state anxiety they felt when playing games was lower. These results corroborated with those of our previous studies [16,18,19]. Elite athletes can control their anxiety during competition, which may lead to better performance. Higher ranking baseball players show increased brain activation in response to baseball errors, indicating that they are controlling their anxiety [19]. Esports players and pro-baseball players in particular can control their own anxiety well in certain situations. This allows them to feel less uneasiness and nervousness, and thus perform better in season games or other important games, such as rivalry matches or finals, in front of huge audiences.

In the present study, esports players were significantly faster on the Tower of London test and performed better on the Mental Rotation test, though there were no differences in the correction rate on the Tower of London test. This means that esports players may need lesser time to solve problems than the other groups. The faster reaction times in the Tower of London test in esports players may also indicate that they have a more economic use of working memory when planning and manipulating temporarily retained information [20,41]. As esports players are faster than the general population and also pro-baseball players in this regard, it is likely that working memory is crucial to improve internet gaming skills. The better performance in the Mental Rotation test suggests that esports players have excellent spatio-temporal cognitive abilities, and that this ability is important in gaming skills as well [22,42].

Pro-baseball players performed significantly faster in the Emotional Perception and Mental Rotation tests. Their faster reaction times in these two tests suggest that they have superior intuitive perception. We can infer that intuitive perception is a key characteristic to become a professional baseball player [43].

When considering the correlation between psychological and cognitive characteristics, high self-transcendence scores were positively correlated with the reaction times on the Tower of London test in esports players and with the reaction times on the Emotional Perception test in pro-baseball players. As mentioned above, high self-transcendence can be associated with developing one’s skills to the professional level, both in esports and baseball [44]. Taken together, these results suggest that esports players may depend more on the cognitive domain, while professional baseball players may depend on the emotional (intuitive) domain, when working to improve their skills. With enhanced cognitive functioning, esports players may be better able to react in response to avatar or object stimulation while pro-baseball players may be better able to react to human movement. We speculate that these differences may lead to the improvement of intuitive perception in pro-baseball players but not in esports players.

### 4.2. Comparison of Psychological and Cognitive Characteristics between the Elite and General Groups

When comparing the elite and non-elite groups, the elite esports and pro-baseball players showed higher novelty seeking, self-directedness, and self-transcendence scores than the non-elite groups, just as the whole esports and pro-baseball groups showed higher scores than the general population. Likewise, the elite esports players showed lower state anxiety than the non-elite esports players group, as well as faster reaction times on the Tower of London and Mental Rotation tests, and better performance on the Mental Rotation test.

Interestingly, the self-transcendence score of the general esports players was lower than that of the general population. Given the correlation between self-transcendence scores and reaction times on the Tower of London test in esports players, the self-transcendence score could be a predictive factor for better performance in working memory in esports play.

Elite pro-baseball players showed faster reaction times on the Emotional Perception test and Mental Rotation test than the non-elite pro-baseball players. The important psychological and cognitive characteristics that we identified can be used to distinguish elite from non-elite professionals as well as professionals from the general population.

### 4.3. Limitations

This study had several limitations. First, the sample size was too small to generalize our findings to all esports and pro-baseball players. Second, we should consider reporting bias, as the TCI and STAI-KY data are fundamentally based on self-report questionnaires. Players differ in their self-evaluation strictness. Further study is needed in this regard. Third, because the current study was a cross-sectional case control study, we are unable to determine whether the increased abilities of esports players are intrinsic or learned abilities. Future research should include longitudinal cohort studies to examine the changes in psychological and cognitive abilities in response to practice in esports players. Together with current results, a longitudinal study would be able to show the effects of internet game play on psychological and cognitive changes in internet game users.

## 5. Conclusions

Like pro-baseball players, esports players should be considered professionals in terms of their psychological and cognitive abilities. Advanced cognitive functions and well-controlled emotion regulation in esports players has previously been reported in many studies [5,6,7,8,9,10,11]. Current results replicated and extended those findings by comparing esports players to professional baseball players. Esports players and pro-baseball players have similar temperament characteristics but different cognitive characteristics; enhanced cognitive abilities were associated with superior performance in esports players while enhanced intuitive abilities were associated with superior performance in pro-baseball players. This intuitive perception was speculated to be unique to becoming an elite professional baseball player [43].

## Figures and Tables

**Table 1:** Demographic characteristics.

	Pro-Gamers (*n* = 55)		Pro-Baseball (*n* = 57)		HC (*n* = 60)	Statistics
**Age** **(mean ± SD)**	21.3 ± 1.4		21.3 ± 1.4		21.3 ± 1.5	*F* = 0.01, *p* = 0.99, ŋ = 0.004
**Education** ** (years) (mean ± SD)**	12.1 ± 0.8		11.9 ± 0.2		12.1 ± 1.3	*F* = 0.51, *p* = 0.60, ŋ = 0.203
**Career** **(years) (mean ± SD)**	3.3 ± 1.1		3.3 ± 1.4		-	*t* = 0.06, *p* = 0.95, *d* < 0.001
**Genre/Position**	Overwatch	16 (29.0)	Infield	21 (36.8)	-	
***n*** **(%)**	BG	7 (12.7)	Outfield	12 (21.1)	-	
	LOL	20 (36.4)	Pitcher	24 (42.1)	-	
	StarCraft	12 (21.9)				

SD: standard deviation; BG: Battleground; LOL: League of Legends; HC: healthy comparison subjects; ŋ: partial eta-squared, *d*: Cohen’s d.

**Table 2:** Psychological and cognitive characteristics of all participants.

	Pro-Gamers (*n* = 55)	Pro-Baseball (*n* = 57)	HC (*n* = 60)	Statistics
**Psychological Characteristics**				
**Temperament and Characteristics Inventory (Mean** **± Standard Deviation)**				
NS ***	21.5 ± 5.4	21.6 ± 4.9	18.7 ± 3.7	*F* = 8.99, *p* < 0.01, ŋ = 0.818
HA ***	18.7 ± 6.3	13.9 ± 7.1	14.6 ± 4.9	*F* = 6.29, *p* < 0.01, ŋ = 0.759
RD	14.4 ± 4.0	15.1 ± 3.1	15.2 ± 3.2	*F* = 0.63, *p* = 0.53, ŋ = 0.239
P	5.0 ± 2.0	5.5 ± 1.7	4.8 ± 1.6	*F* = 2.89, *p* = 0.06, ŋ = 0.591
SD **	24.1 ± 5.9	24.9 ± 4.7	21.8 ± 6.1	*F* = 6.26, *p* < 0.01, ŋ = 0.757
C	27.4 ± 4.8	29.2 ± 6.1	27.3 ± 5.5	*F* = 2.07, *p* = 0.13, ŋ = 0.509
ST **	15.6 ± 5.9	15.1 ± 4.9	12.9 ± 5.4	*F* = 11.69, *p* < 0.01, ŋ = 0.853
**State-Trait Anxiety Inventory-KY (Mean** **± Standard Deviation)**				
State *	37.7 ± 9.4	37.2 ± 8.7	42.4 ± 5.6	*F* = 8.41, *p* < 0.01, ŋ = 0.808
Trait	39.2 ± 10.2	38.2 ± 10.4	40.8 ± 6.1	*F* = 1.52, *p* = 0.22, ŋ = 0.432
**Neurocognitive Function Tests**				
**Modified Tower of London (Mean** **± Standard Deviation)**				
ToL-RT *	6.7 ± 1.0	8.3 ± 2.1	8.4 ± 1.9	*F* = 9.31, *p* < 0.01, ŋ = 0.823
ToL-CR	7.1 ± 0.7	7.1 ± 0.8	7.6 ± 1.1	*F* = 0.31, *p* = 0.42, ŋ = 0.134
**Emotional Perception (Mean** **± Standard Deviation)**				
EP-RT *	4.3 ± 1.2	3.6 ± 0.9	4.5 ± 1.2	*F* = 8.62, *p* < 0.01, ŋ = 0.812
EP-CR	80.1 ± 7.1	75.4 ± 10.8	74.9 ± 9.7	*F* = 2.91, *p* = 0.06, ŋ = 0.592
**Mental Rotation (Mean** **± Standard Deviation)**				
MR-RT *	3.6 ± 0.9	2.9 ± 1.2	3.7 ± 1.0	*F* = 9.67, *p* < 0.01, ŋ = 0.829
MR-CR *	88.8 ± 4.8	70.1 ± 15.9	70.7 ± 13.9	*F* = 19.63, *p* < 0.01, ŋ = 0.907

HC: healthy comparison subjects; NS: novelty seeking; HA: harm avoidance; RD: reward dependence; P: persistence; SD: self-directedness; C: cooperativeness; ST: self-transference; ToL: modified Tower of London; Korean version of the State-Trait Anxiety Inventory-Y (STAI-KY), EP: Emotional Perception test; MR: Mental Rotation test; RT: reaction time; CR: correction rate; ***: statistically significant (0.013); **: statistically significant (0.017); *: statistically significant (0.025); ŋ: partial eta-squared; Post hoc (*p* < 0.05); NS: pro-gamers = pro-baseball > HC; HA: pro-baseball = HC < pro-gamers; SD: pro-gamers = pro-baseball > HC; ST: pro-gamers = pro-baseball > HC; State: pro-gamers = pro-baseball < HC; ToL-RT: pro-gamers < pro-baseball = HC; EP-RT: pro-baseball < HC = pro-gamers; MR-RT: pro-baseball < HC = pro-gamers; MR-CR: pro-gamers > pro-baseball = HC.

**Table 3:** Comparison of psychological and cognitive characteristics between elite pro-gamers and general pro-gamers.

	Elite Pro-Gamers (*n* = 12)	General Pro-Gamers (*n* = 43)	Statistics
**Psychological Characteristics**			
**Temperament and Characteristics Inventory (Mean** **± Standard Deviation)**			
NS ***	27.6 ± 3.1	18.4 ± 3.2	*t* = 6.00, *p* < 0.01, *d* = 2.761
HA	18.0 ± 6.7	19.1 ± 6.2	*t* = −0.53, *p* = 0.43, *d* = 0.170
RD	14.9 ± 3.4	14.1 ± 4.4	*t* = 0.68, *p* = 0.49, *d* = 0.203
P	4.8 ± 2.3	5.2 ± 1.9	*t* = −1.54, *p* = 0.13, *d* = 0.189
SD **	29.6 ± 3.7	22.6 ± 5.6	*t* = 3.06, *p* < 0.01, *d* = 1.475
C	27.4 ± 3.2	27.4 ± 5.6	*t* = 0.06, *p* = 0.82, *d* < 0.001
ST **	17.3 ± 5.1	8.4 ± 3.2	*t* = 4.82, *p* < 0.01, *d* = 2.107
**State-Trait Anxiety Inventory-KY (Mean** **± Standard Deviation)**			
State *	31.1 ± 4.4	41.2 ± 9.6	*t* = −3.19, *p* < 0.01, *d* = 1.353
Trait	39.3 ± 7.1	39.2 ± 11.7	*t* = 0.08, *p* = 0.64, *d* = 0.010
**Neurocognitive Function Tests**			
**Modified Tower of London (Mean** **± Standard Deviation)**			
ToL-RT *	5.9 ± 0.4	7.1 ± 1.0	*t* = −3.03, *p* < 0.01, *d* = 1.576
ToL-CR	7.1 ± 0.9	7.2 ± 0.6	*t* = −0.09, *p* = 0.76, *d* = 0.131
**Emotional Perception (Mean** **± Standard Deviation)**			
EP-RT *	3.4 ± 0.8	4.7 ± 1.2	*t* = −2.08, *p* = 0.01, *d* = 1.274
EP-CR	83.1 ± 5.4	78.5 ± 5.6	*t* = 1.72, *p* = 0.06, *d* = 0.836
**Mental Rotation (Mean** **± Standard Deviation)**			
MR-RT	3.7 ± 0.9	3.6 ± 0.9	*t* = 0.56, *p* = 0.48, *d* = 0.111
MR-CR *	93.0 ± 3.2	86.5 ± 3.9	*t* = 4.33, *p* < 0.01, *d* = 1.822

NS: novelty seeking; HA: harm avoidance; RD: reward dependence; P: persistence; SD: self-directedness; C: cooperativeness; ST: self-transference; ToL: modified Tower of London; EP: Emotional Perception test; MR: Mental Rotation test; RT: reaction time; CR: correction rate; ***: statistically significant (0.013); **: statistically significant (0.017); *: statistically significant (0.025), *d*: Cohen’s d.

**Table 4:** Comparison of psychological and cognitive characteristics between elite pro-baseball players and general pro-baseball players.

	Elite Pro-Baseball (*n* = 14)	General Pro-Baseball (*n* = 43)	Statistics
**Psychological Characteristics**			
**Temperament and Characteristics Inventory (Mean** **± Standard Deviation)**			
NS ***	25.5 ± 4.4	19.9 ± 4.1	*t =* 3.97, *p <* 0.01, *d =* 1.316
HA	14.5 ± 6.4	13.7 ± 7.3	*t =* 1.22, *p =* 0.17, *d =* 0.116
RD	14.4 ± 3.8	15.3 ± 2.9	*t =* −1.44, *p =* 0.13, *d =* 0.266
P	5.3 ± 1.7	5.6 ± 1.7	*t =* −1.76, *p =* 0.08, *d =* 0.176
SD **	29.9 ± 3.1	23.1 ± 3.8	*t =* 3.21, *p <* 0.01, *d =* 1.961
C	27.3 ± 7.7	29.9 ± 5.4	*t =* −1.66, *p =* 0.08, *d =* 0.391
ST **	14.8 ± 4.9	11.2 ± 4.2	*t =* 3.34, *p =* 0.01, *d =* 0.788
**State-Trait Anxiety Inventory-KY (Mean** **± Standard Deviation)**			
State	32.2 ± 6.2	38.9 ± 8.9	*t =* 2.96, *p =* 0.03, *d =* 0.873
Traits	38.8 ± 13.6	38.6 ± 9.3	*t =* 0.07, *p =* 0.66, *d =* 0.069
**Neurocognitive Function Tests**			
**Modified Tower of London (Mean** **± Standard Deviation)**			
ToL-RT	8.2 ± 1.4	8.4 ± 2.3	*t =* −1.43, *p =* 0.14, *d =* 0.105
ToL-CR	7.5 ± 0.7	7.3 ± 0.9	*t =* 1.49, *p =* 0.12, *d =* 0.248
**Emotional Perception (Mean** **± Standard Deviation)**			
EP-RT *	2.9 ± 0.8	3.8 ± 0.8	*t =* −3.11, *p <* 0.01, *d =* 1.125
EP-CR	76.2 ± 11.8	75.2 ± 10.6	*t =* 1.77, *p =* 0.08, *d =* 0.089
**Mental Rotation (Mean** **± Standard Deviation)**			
MR-RT	2.6 ± 0.8	3.0 ± 1.3	*t =* 2.97, *p =* 0.03, *d =* 0.371
MR-CR	67.8 ± 11.7	70.8 ± 17.1	*t =* 1.87, *p =* 0.08, *d =* 0.205

NS: novelty seeking; HA: harm avoidance; RD: reward dependence; P: persistence; SD: self-directedness; C: cooperativeness; ST: self-transference; ToL: modified Tower of London; EP: Emotional Perception test; MR: Mental Rotation test; RT: reaction time; CR: correction rate; ***: statistically significant (0.013); **: statistically significant (0.017); *: statistically significant (0.025), *d*: Cohen’s d.
